# Diagnosis and Management of Inadvertent Iliac Vein Injury During Structural Heart Interventions

**DOI:** 10.1016/j.jaccas.2024.103096

**Published:** 2025-02-05

**Authors:** Ruxandra I. Sava, Sandra Zendjebil, Maximilien Sochala, Morad Djebbar, Noemie Tence, Philippe Garot

**Affiliations:** Interventional Cardiology Department, Institut Cardiovasculaire Paris-Sud, Hôpital privé Jacques Cartier, Ramsay Santé, Massy, France

**Keywords:** bleeding, iatrogenic vein perforation, outcomes, stent

## Abstract

Femoral venous access is increasingly used by structural interventional cardiologists in procedures such as patent foramen ovale, atrial septal defect, and left atrial appendage closure, as well as tricuspid and mitral interventions. Containing less muscular and elastic fibers than arteries, veins are fragile structures that are more easily pierced than arteries. In this case series, we present 2 cases of iliac vein perforation during structural cardiology interventions. Even mild resistance on advancement of a large sheath should prompt suspicion of venous perforation, in which case control angiography should be performed at the end of the procedure, after withdrawal of the sheath distal to the suspected perforation site. In case of venous breach, implantation of a peripheral artery–dedicated covered stent can efficiently seal the perforation. This forethought can allow the avoidance of severe complications such as retroperitoneal hemorrhage.

## Visual Summary

**Figure:**
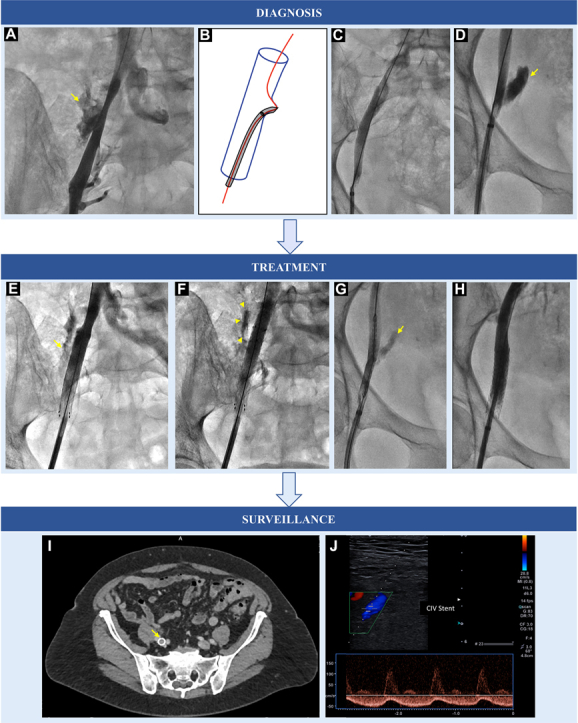

**Visual Summary:**
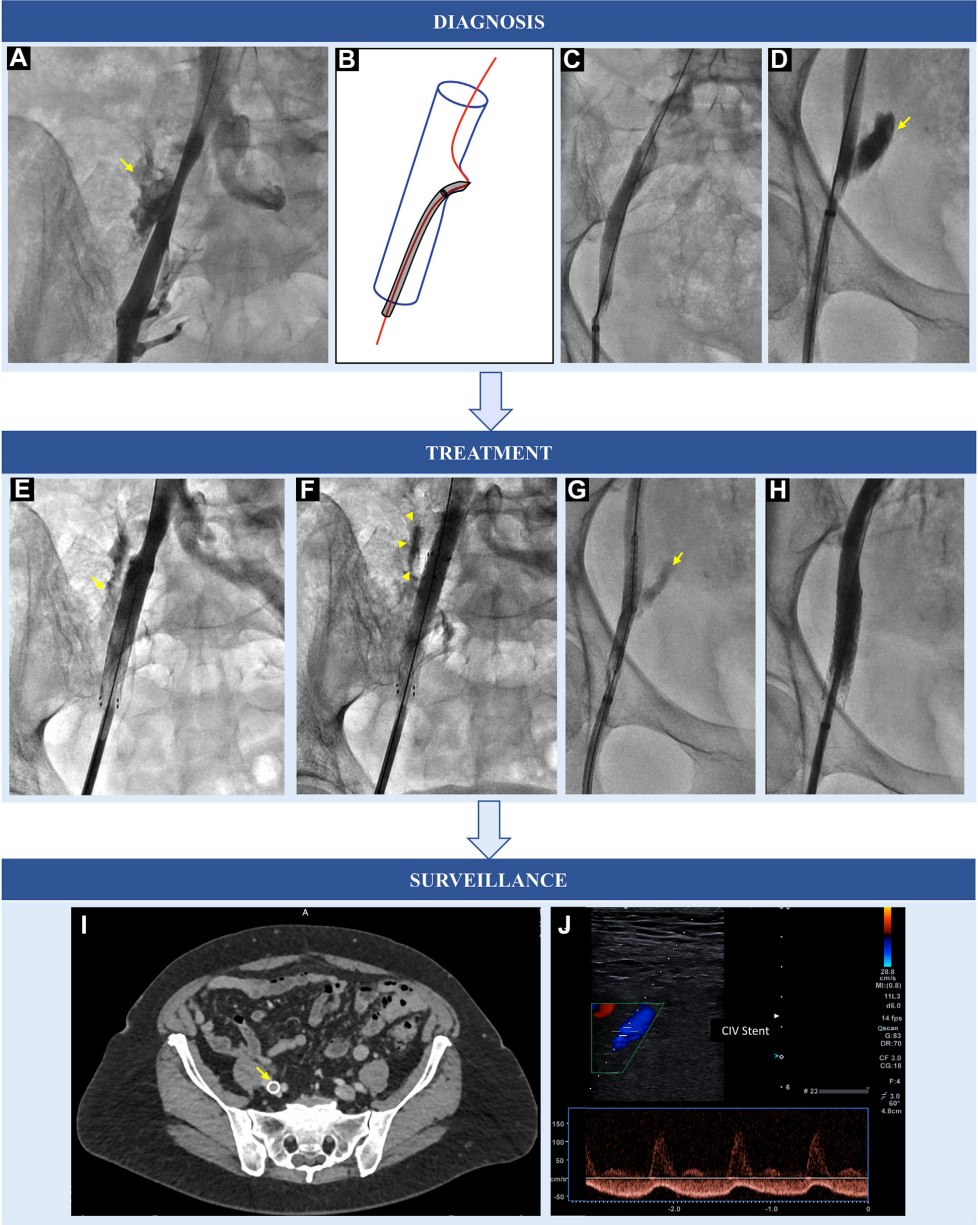
Diagnosis and Management of a Venous Perforation (A) Case 1. After feeling resistance on advancement of a 12-F introducer sheath, contrast injection performed at the end of the procedure revealed perforation of the common iliac vein. (B to D) Case 2. Resistance was felt during the introduction of the 9-F sheath and dilator. (B) Drawing depicting findings of fluoroscopy, showing the guidewire and sheath pointing out of the venous contour. (C) Initial phlebography performed with the proximal end of the sheath proximal to the site of bleeding revealed a false-negative result, with no bleeding. (D) Slight retraction of the sheath, distal to the site of bleeding, allowed visualization of contrast extravasation (arrow). (E and F) Case 1. (E) Control phlebography after covered stent implantation (10 × 60-mm self-expandable covered stent) revealed persistent contrast extravasation, suggesting inadequate sealing by the stent (arrow). (F) Implantation of a second stent (10 × 37-mm balloon-expandable covered stent) proximally to the first and with a small area of overlap between the 2 stents successfully stopped the bleeding. Note contrast stagnation in the tissues surrounding the stent (arrowheads). (G and H) Case 2. (G) Positioning of the covered stent overlying the venous breech. (H) An adequately positioned 10 × 57-mm balloon-expandable stent successfully stopped the bleeding. (I) Case 1. Computed tomography surveillance performed 1 year later revealed a patent iliac stent (arrow). (J) Case 2. Doppler venous ultrasonography performed 1-week postprocedure because of abdominal pain showed a patent stent and no evidence of activebleeding.

Apart from transcatheter aortic valve replacement (TAVR), most structural cardiology interventions involve mid or large bore femoral vein access. In a study analyzing retrieved data from the Agency for Healthcare Research and Quality's Healthcare Cost and Utilization Project National Inpatient Sample, left atrial appendage closure and transcatheter edge-to-edge repair represented 29% and TAVR 71% of structural cardiology interventions performed in the United States from January 2016 to December 2020.^1^ Although most operators exercise increased caution while navigating the femoral and iliac arteries during TAVR sheath placement, using stiff guidewires and continuous fluoroscopic guidance to avoid excessive interaction with the arterial wall, venous sheaths are usually advanced with less precautions. However, the walls of the iliofemoral veins are much thinner than those of arteries, containing less muscular and elastic fibers. Thus, venous perforation is more likely than arterial perforation even with mild forceful advancement of large introducer sheaths.

**Take-Home Messages**

• Veins are fragile structures, and any resistance encountered during advancement of a long, large sheath should prompt fluoroscopic control. A high index of suspicion for perforation is needed in case of any abnormal bend of the guide–sheath complex.
• If perforation is suspected, the large sheath will prevent bleeding during the procedure. The sheath should be retracted and then gently readvanced under fluoroscopy, allowing continuation of the main procedure.
• At the end of the procedure, the guidewire should be kept in the venous lumen, and contrast injection should be performed after retraction of the sheath distal to the site of suspected perforation. In the event of bleeding, a covered peripheral vascular stent should be implanted to achieve hemostasis.

## Case 1

### History of presentation

A 77-year-old woman presented with moderate dyspnea on exertion, occasional chest pain on exertion, and repeated bouts of syncope. The patient presented no cardiovascular risk factor other than age and being overweight.

### Diagnostic workup

The electrocardiogram showed atrial flutter and incomplete right bundle branch block. Standard blood tests were normal. The transthoracic echocardiogram showed normal left ventricular function, an atrial septum defect (ASD), moderate right ventricular and right atrial dilatation, and significant right–left shunt with the Q_p_/Q_s_ calculated at 2.1. The transesophageal echocardiogram (TEE) confirmed these findings, showing a multiperforated, secundum-type interatrial septum defect coupled with an interatrial septum aneurysm. Cardiac magnetic resonance imaging confirmed these findings, with a Q_p_/Q_s_ of 1.5 and no additional cardiac anomalies. Coronary angiography showed patent coronary arteries with no significant stenosis. Considering these findings, age of the patient, and local expertise, the heart team decided in favor of interventional ASD closure.

### Interventions

Atrial flutter was treated by ablation of the cavotricuspid isthmus. Interventional ASD closure was performed using angiographic and TEE guidance. The procedure was conducted using a right femoral vein approach, using a 9-F introducer sheath (RadiFocus, Terumo). Immediately after preclosing (PROGLIDE, 6-F, Abbott), we administered full-dose heparin (100 U/kg). A standard 260-cm 0.035-inch guidewire was negotiated into the right atrium, over which the 9-F sheath was exchanged for a 12-F sheath (Check-Flo Introducer, Cook).

At this time, we noted resistance on sheath advancement at the level of the right iliac vein. We therefore slightly retracted the sheath, reoriented the dilator tip, and successfully navigated through the tortuosity under fluoroscopic guidance. At this time, we considered the possibility of venous breach, but we decided to continue the procedure because we expected the 12-F sheath to prevent bleeding through internal compression, and the patient showed no sign of hemodynamic compromise. The ASD was crossed using a 0.035-inch guidewire through a JR4 5-F diagnostic catheter (DxTerity, Medtronic) and efficiently closed by implantation of a 35-mm Septal Occluder Cribriform prosthesis (Abbott Cardiovascular), with no residual shunt.

At the end of the procedure, contrast injection revealed perforation of the common iliac vein (Video 1). We thus decided to implant 10 × 60-mm self-expandable covered stent (Fluency Plus, Bard Medical) that was postdilated using a 12 × 40-mm peripheral percutaneous transluminal angioplasty balloon catheter (Oceanus, iVascular) inflated at 6 atm for 60 seconds. On control phlebography, we noticed the first covered stent did not completely seal the perforation (Video 2); thus, we decided to implant a second stent, distal to and with a small area of overlap with the first stent. We opted for a 10 × 37-mm balloon-expandable covered stent (iCover, iVascular) inflated at 9 atm for 60 seconds. This efficiently stopped the bleeding, with no leak at the end of the procedure (Video 3).

### Outcomes

A same-day abdominal computed tomography showed that the stents implanted at the level of the right iliac vein were patent, and no contrast extravasation was noted. The patient was asymptomatic and discharged the day after the procedure, with no hemoglobin drop or any other complication. She was prescribed oral anticoagulant treatment (apixaban 5 mg × 2/day) in view of her previous atrial flutter diagnosis and CHA_2_DS_2_-VASc score of 3. At the 16-month follow-up, the patient was asymptomatic, and a repeat abdominal computed tomography revealed a patent stent.

## Case 2

### History of presentation

A 66-year-old woman with a history of well-controlled hypertension and mild dyslipidemia presented for sudden onset of hemiparesis and paresthesia of the left lower limb.

### Diagnostic workup

The cerebral magnetic resonance imaging diagnosed an acute ischemic stroke of the right Rolandic operculum. The etiological workup revealed absence of atrial fibrillation, intracardiac thrombus, or significant stenosis of the supra-aortic arteries. However, TEE revealed a large patent foramen ovale (PFO) associated with an aneurysm of the interatrial septum. This was considered the likely cause of her stroke, and she was referred to our center for interventional closure.

### Interventions

Interventional PFO closure was performed using angiographic and TEE guidance, using right femoral vein approach. The procedure followed the same main steps as described in Case 1. Based on the TEE findings, a decision was made to implant a 30/25-mm prosthesis (Amplatzer Talisman, Abbott), which was introduced through a 9-F sheath (Amplatzer Talisman Delivery sheath, Abbott). While advancing the sheath and dilator over a standard 0.035-inch standard guidewire, resistance was felt, and fluoroscopy revealed the relatively floppy guidewire did not efficiently navigate the venous tortuosity. Both the guidewire and the sheath were pointing outward of the venous contour.

Having suspected a venous perforation, after successful PFO occlusion and withdrawal of the sheath distal to the level of the suspected venous perforation, we performed a diagnostic contrast injection. At this time, care must be taken not to perform contrast injection proximally to the lesion, because this will result in a false-negative result (Video 4). Indeed, a more distal injection revealed venous perforation (Video 5). We thus decided to implant a 10 × 57-mm balloon-expandable covered stent (iCover, iVascular) at 12 atm for 60 seconds. Control contrast injection distal to the stent revealed adequate vascular sealing (Video 6).

### Outcomes

The patient was discharged on the same day, without postprocedural hemoglobin drop or other complications. She was prescribed 160 mg aspirin a day for 1 year. One week later, she complained of mild right lower quadrant pain. Venous Doppler ultrasonography revealed a patent stent, without signs of active bleeding and with minimal hematoma surrounding the vein, which likely explained the abdominal pain. At this time, the hemoglobin level was unchanged, and the patient did not require hospitalization. At a telephone follow-up 6 months later, the patient was asymptomatic and did not require further medical attention.

## Discussion

These clinical cases illustrate the great veins are vulnerable to perforation on advancement of a large-caliber introducer sheath on a standard 0.035-inch guidewire that has become bent or kinked, even with mild pressure. Both the 9-F and the 12-F introducers were effective in sealing the venous perforation during the procedure, and both 10- and 12-mm arterial periphery–dedicated covered stents adequately sealed the perforation after introducer removal. Moreover, we focused on achieving rapid hemostasis and did not attempt therapeutic balloon inflation for these overt, major venous breaches. Care must be taken to choose covered stents that are long enough, as illustrated by our first case in which the first stent was too short, leading to geographic miss and requirement for a second covered stent. Adequately sized balloon-expandable and self-expandable stents were equally capable of sealing the venous breach.

Despite the low pressures of the venous system, breach of a large vein can cause hemorrhagic shock, with estimated mortality rates ranging from 6% to 22%.^2^ The onset of shock will depend on perforation size. Although a small breach may cause hemodynamic compromise after conclusion of the procedure, prompting additional diagnostic tests for diagnosis and requiring a second venous puncture for treatment, a large perforation may rapidly cause hemorrhagic shock. This is illustrated by the case report published by Landolff et al^3^ in which an unsuspected massive perforation of the right external iliac vein arising during a left atrial appendage closure procedure was complicated by shock immediately after retraction of the venous sheath. Although implantation of 2 covered stents adequately sealed the perforation, the patient required vasopressor therapy, a blood transfusion, and hospitalization in the intensive care unit.

The challenge with venous perforation lies not in the treatment but in the accurate recognition of the perforation before major blood loss has occurred. Suspicion of this complication should prompt a diagnostic phlebography immediately after retraction of the sheath distal to the expected location of the breach. Moreover, 10- and 12-mm diameter covered stents should be available during structural cardiology procedures conducted using a venous approach.

Considering the evolving indications for procedures such as left atrial appendage closure and transcatheter edge-to-edge repair, venous access will be used by an increasing number of operators. Patient-related factors, such as marked venous tortuosity, and operator-related factors, such as proximal femoral vein puncture, may increase the risk of venous injury. Strategies to reduce this risk include placement of large sheaths under fluoroscopic guidance and tracking over stiff 0.035-inch guidewires, which offer better support for introducer advancement. Because venous tortuosity is difficult to anticipate, we now systematically use a stiff guidewire (specifically the Amplatzer Super Stiff) for venous introducer sheath advancement.

Finally, our clinical practice includes systematic closure of midbore venous punctures using vascular closure devices, specifically suture-based systems. This practice is supported by a study that compared manual compression with vascular closure devices in patients undergoing atrial fibrillation ablation or left atrial appendage occlusion, which found that vascular closure devices were associated with faster hemostasis, less bleeding complications, and increased patient satisfaction.^4^

## Conclusions

Veins are fragile structures, and the use of standard 0.035-inch guidewires can cause difficulties while navigating tortuous anatomy. This, combined with the rigidity and caliber of the introducer sheets used for structural cardiology interventions requiring venous access, can cause vascular perforation even with mild forceful advancement. Although bleeding during the procedure will likely be prevented by the large French sheath, suspicion of venous injury should prompt diagnostic phlebography before concluding the procedure, allowing early identification of venous injury. Adequately sized covered stents are effective in sealing venous perforations and should be available in laboratories performing structural interventions. Suspicion of a venous perforation based on procedural events is key, because a late diagnosis after overt hemorrhagic shock is associated with increased mortality.

## Funding Support and Author Disclosures

Dr Sava was supported by an unrestricted fellowship grant from The European Association of Percutaneous Cardiovascular Interventions, sponsored by Edwards. All other authors have reported that they have no relationships relevant to the contents of this paper to disclose.

## Appendix

Video 1 Case 1: Contrast extravasation seen after venous sheath retraction.

Video 2 Case 1: Contrast injection after placement of first covered stent revealed persistent bleeding.

Video 3 Case 1: Control injection after placement of the second covered stent revealed adequate sealing of bleeding.

Video 4 Case 2: Bleeding not visible on phlebography because of insufficient retraction of the venous sheath.

Video 5 Adequate visualization of bleeding after additional retraction of the venous sheath.

Video 6 Case 2: Bleeding stopped after adequate implantation of 1 covered stent at the site of the venous perforation.
